# mSphere of Influence: the Mycobiota in Human Health and Disease

**DOI:** 10.1128/mSphere.00974-19

**Published:** 2020-01-22

**Authors:** Soo Chan Lee

**Affiliations:** a South Texas Center for Emerging Infectious Diseases (STCEID), Department of Biology, University of Texas at San Antonio, San Antonio, Texas, USA

**Keywords:** microbiota, mycobiome, mycobiota, mycology, pathogenic fungi

## Abstract

Soo Chan Lee works in the field of medical mycology. In this mSphere of Influence article, he reflects on how “Interactions between commensal fungi and the C-type lectin receptor Dectin-1 influence colitis” (Science 336:1314–1317, 2012, https://doi.org/10.1126/science.1221789) by I. D. Iliev, V. A. Funari, K. D. Taylor, Q. Nguyen, et al., “CX3CR1^+^ mononuclear phagocytes control immunity to intestinal fungi” (Science 359:232–236, 2018, https://doi.org/10.1126/science.aao1503) by I. Leonardi, X. Li, A. Semon, D. Li, et al., and “The fungal mycobiome promotes pancreatic oncogenesis via activation of MBL” (Nature 574:264–267, 2019, https://doi.org/10.1038/s41586-019-1608-2) by B. Aykut, S. Pushalkar, R. Chen, Q. Li, et al. made an impact on him to study medically important fungi by providing a forum to understand the roles of fungal microbiota or mycobiota in human diseases and health.

## Abstract (precis)

Soo Chan Lee works in the field of medical mycology. In this mSphere of Influence article, he reflects on how “Interactions between commensal fungi and the C-type lectin receptor Dectin-1 influence colitis” (Science 336:1314–1317, 2012, https://doi.org/10.1126/science.1221789) by I. D. Iliev, V. A. Funari, K. D. Taylor, Q.

## COMMENTARY

Fungi are eukaryotic organisms that belong to the clade *Opisthokonts*, which encompasses both the animal and fungus kingdoms. Fungi play key roles in the ecosystem as natural decomposers and in the production of foods. They are also valuable in industry as producers of biofuels such as biodiesel and ethanol and even medicines. Fungi have also served as a model system for understanding many aspects of biology. In addition, a group of fungi can infect humans and cause diseases. Fungal infections have increased in recent decades and now pose a serious threat to public health as the number of susceptible cohorts such as immunocompromised patients has increased. During my postdoctoral training, I have therefore focused my research on human-pathogenic fungi to study how they interact with host cells and the fungal virulence factor genes that are involved in these interactions. In addition, my research has also involved fungal morphogenesis in pathogenesis and elucidation of the mechanisms of antifungal drug resistance.

My understanding of medically important fungi has grown through reading the study by Iliev et al. (1) on the fungal microbiome, generally called the mycobiome. This study verified that failure to recognize intestinal fungi in mice with mutations in the gene encoding Dectin-1 results in the exacerbation of colitis compared to cohorts of mice lacking these mutations. They revealed that Dectin-1 mutant mice have an elevated susceptibility to chemically (dextran sodium sulfate [DSS]) induced colitis. When DSS was used to elicit colitis in mice with this mutation, the abundance of the opportunistic pathogenic fungus Candida tropicalis in the gut increased and the fungus invaded inflamed tissues, whereas in DSS-treated wild-type mice, the fungus stayed in the gut lumen. Furthermore, they verified that a single nucleotide polymorphism (SNP) in the Dectin-1 gene is linked to the severity of human ulcerative colitis. Dectin-1 is a pattern recognition receptor (PRR) and participates in the immune recognition of fungi, especially via the major fungal cell wall component beta-glucan (2). This study therefore elucidated that intestinal fungi influence gastrointestinal (GI) tract diseases. Another study by Leonardi et al. (3) further verified that the fracktalkine receptor 1 (CX3CR1) of the hosts plays a key role during interaction with intestinal fungi. This study found that when mice were colonized with the commensal fungus Candida albicans, the CX3CR1^+^ mononuclear macrophages played a key role in intestinal antifungal immunity. Importantly, their study further demonstrated that SNPs in the gene encoding CX3CR1 are strongly associated with IgG responses against fungi in patients with Crohn’s diseases. These studies suggest that enteric fungi are also key components as well as bacteria in the gut microbiota that affect human health and disease. Another commensal dandruff fungus, *Malassezia* species, was found to affect pancreatic ductal adenocarcinoma (PDA) oncogenesis via another PRR mannose binding lectin (MBL) that recognizes glycan in the fungal cell wall (4). The abundance of *Malassezia* spp. was significantly increased in the PDA tissues compared to normal pancreatic tissues, and the fungus was found to infiltrate from the GI tract to the pancreas.

Human microbiota are a collection of microbes in a certain site or habitat, and the term microbiome refers to the catalog of these of microbes and their genetic material (5). Recent studies have elucidated the role of human gut microbiota in many human diseases, ranging from inflammatory bowel disease, obesity, and cancer to traumatic brain injuries and even to anorexia nervosa (6–10). Numerous studies have identified detrimental bacterial species associated with specific diseases and beneficial bacteria to improve gut health. Interactions between bacterial species have also been identified. Studies have also found that dysbiosis in bacterial components is associated with these diseases. Reconstruction of the microbiota has been considered as a therapeutic option in many diseases.

However, in these studies of the microbiota, fungi have been neglected even though the evidence suggests that fungi are also strongly associated with human health and disease (11–13). Indeed, the fungus *Saccharomyces boulardii* is one of the early probiotics used to improve GI tract health. For example, antibiotic-driven dysbiosis can be relieved by ingestion of this fungus (14). Anti-*Saccharomyces* antibodies have been used as a serological marker for inflammatory bowel disease (15). Studies therefore strongly support the role of intestinal fungi in disease. The aforementioned studies further revealed that fungi in the gut play key roles in inflammation in the gut and even in cancer. It is therefore obvious that enteric fungi clearly play important roles in human health and disease. These studies have deeply influenced my desire to research on medically important fungi as a component of the gut microbiota.
